# The effect of salt-drought stress on the growth and physiological characteristics of *Viola tricolor* seedlings

**DOI:** 10.3389/fpls.2025.1552092

**Published:** 2025-09-15

**Authors:** Wenlan Liu, Mengting Huang, Hong Tang, Rong Han, Yongzhong Luo

**Affiliations:** College of Forestry, Gansu Agricultural University, Lanzhou, China

**Keywords:** *Viola tricolor*, salt stress, drought stress, growth characteristics, physiological characteristics

## Abstract

To elucidate the impact of varying degrees of salt, drought, and their combined stress on the physiological and biochemical attributes of *Viola tricolor* seedlings, a pot culture study was conducted. The experiment utilized *Viola tricolor* seedlings as test materials, segregated into 16 treatments: four levels of NaCl (0%, 0.2%, 0.4%, 0.6%) for salt stress; four increments in soil field capacity (80%, 65%, 50%, 35%) for drought stress; and combinations of these stresses to assess their cumulative effects. A control group was established using 0% NaCl and 80% field capacity water content. Growth metrics and physiological indicators were quantified under each condition. Results indicate that with escalating degrees of salt and drought stresses, height, dry weight of above-ground parts, dry weight of below-ground parts, and root-to-shoot ratio exhibit an incremental pattern initially followed by a decline. Relative water content decreases, while Malondialdehyde (MDA) content, soluble protein (SP) content, soluble sugars (SS) content, and proline (Pro) content ascend. The activity of peroxidase (POD), catalase (CAT), and superoxide dismutase (SOD) peaks before declining. However, POD remains elevated under 0.6% NaCl and 35% field capacity under severely stressed conditions compared to the control. Chlorophyll content shows a similar rising and then falling trend. POD emerges as the primary antioxidant enzyme in *Viola tricolor* seedlings when faced with high-salt and severe drought stress. Fuzzy logic analysis suggests that Viola tricolor seedlings demonstrate partial tolerance to moderate salt-drought stress combinations, indicating some cross-adaptation. Specifically, Viola tricolor seedlings exhibit the highest resistance to salt-drought stress under the treatment of 0.2% NaCl and 65% field capacity.

## 1 Introduction

In the last few decades drastic climatic changes have been observed, probably due to global warming, which has brought about not only a serious environmental threat but also seriously affected the growth of the plants (Charfeddine et al., 2023). Plants are frequently exposed to many extremes such as drought stress, low/high temperature, salt stress, flooding stress, and heavy metal toxicity while growing in nature (Zhang et al., 2021). Among these, salinity droughtand are the major environmental constraints that lead to detrimental effects on a plant’s life and hence crop productivity, particularly in arid and semi-arid regions where they often co-occur (Wang et al., 2024). Both salt stress and drought lead to osmotic stress, increasing the difficulty for plants to absorb water through their roots, resulting in dehydration within the plant and accelerated chlorophyll degradation in leaves, thereby impairing photosynthetic apparatus and reducing the rate of photosynthesis, consequently limiting plant growth (Hesami et al., 2020; Yang et al., 2024b). Salt and drought stresses affect many morphological features and alter the physiological processes that are associated with plant growth and development (Yan et al., 2020; Kim et al., 2019). It was reported that in salt and drought stress, plants can resist and adapt to external stresses by enhancing the activity of active oxygen-scavenging enzymes and increasing the content of osmoprotective substances, among other mechanisms (Li et al., 2023). These interconnected responses share a combination of independent yet interrelated regulatory mechanisms that withstand various environmental stresses throughout the plant’s lifecycle. Plants exhibit cross-adaptation upon experiencing non-lethal adverse environmental stresses (either sustained or intermittent), strengthening their resistance against this specific condition and enabling them to resist other unfavourable environments (Yang et al., 2020). Therefore, the study of plant adaptive mechanisms to multiple stressors is paramount.

*Viola tricolor* L. (Violaceae), also known as wild pansy or heart-sease, belongs to the violet family Violaceae, one of the most famous flowering ornamental plants (Mahmood and Hussain, 2022) thrives in fertile soil that is well-draining and rich in organic matter, with a pH range of 5.4 to 7.4 (Liu and Su, 2023). It is distinguished by its profusion of flower colors and long blooming period, which can fill the gap in early spring outdoor flowers in northern China’s floral display lack. In landscape design, it is commonly utilized as material for flower beds, flower beds, and horticultural scenes (Muhammad et al., 2024). Also, it serves a medicinal purpose, known for its cooling properties, dissipation of blood stasis, and cough relief. The entire plant of *Viola tricolor* is also edible. Research has shown that its petals contain abundant anthocyanins (Ponraj and Shashikanth, 2023), flavonoids and carotenoids, indicating their nutritional and nutraceutical value (Fernandes et al., 2019), Regarding stress resistance, studies on *Viola tricolor* have predominantly focused on single-factor stresses (Słomka et al., 2012; Du et al., 2011; Słomka et al., 2011). Nevertheless, in natural environments, plant growth is influenced by multiple factors simultaneously, thereby emphasizing the significance of studying the impact of salt and drought interactive stress on *Viola tricolor* seedlings’ growth and physiological characteristics.

Therefore, in this study, *Viola tricolor* seedlings were selected. Different concentrations of salt and drought stress and their interactive treatments were applied to explore the changes in growth and physiological indicators under salt-drought stress interaction. The objective was to evaluate the plant’s resistance to salt and drought and determine the tolerance range. This research aims to identify suitable soil salinity and drought levels for *Viola tricolor*, thereby providing a theoretical foundation for understanding the mechanisms underlying plant survival under abiotic stress conditions.

## 2 Materials and methods

### 2.1 Plant materials and treatments

In this experiment, seeds of *Viola tricolor* were used as the test material. The New Era Landscape Company purchased the seeds in Lanzhou, Gansu Province. They were cultivated in the College of Forestry, Gansu Agricultural University laboratory, from April to June 2024. Seeds that were uniform in size and had whole kernels were selected. They were sterilized with 2% NaClO for 10 minutes and washed 3-4 times with distilled water. The seeds were then sown in plastic pots containing 400g of air-dried substrate (pot diameter: 16 cm, pot height: 13.8 cm). The substrate, named “Zhuangmiao No.1,” was purchased from Gansu Green Energy Agricultural Technology Co., Ltd. The physicochemical properties of the soil were as follows: pH 6.3, total carbon 85.734 mg·g^-^¹, total nitrogen content 6.736 mg·g^-^¹, total phosphorus 0.797 mg·kg^-^¹, soil bulk density 1.537 g·cm^-^³, and field water holding capacity 75.5%. After thoroughly watering, the pots were placed in a greenhouse to allow for natural germination and growth, managed under standard conditions. Approximately 15 seedlings with consistent growth were retained in each pot, totalling 48 pots. When the seedlings reached 45 days of growth, stress treatment was initiated.

A randomized block design incorporated 16 salt and drought stress treatments. This included four individual salt gradients (with NaCl content in the soil constituting 0%, 0.2%, 0.4%, and 0.6% of the air-dry soil weight, representing control S0, mild stress S1, moderate stress S2, and severe stress (where soil moisture content accounted for 80%, 65%, 50%, and 35% of the field capacity, corresponding to actual soil moisture levels of 60.4%, 49.1%, 37.8%, and 26.4%, respectively denoting normal watering D0, mild stress D1, moderate stress D2, and severe stress D3). Additionally, there were nine combined salt and drought stress treatments (random combinations of S1, S2, S3 with D1, D2, D3), with S0D0 serving as a control group. Each treatment was replicated three times. The soil salinity was controlled by dissolving NaCl in distilled water to prepare solutions of the appropriate concentrations for irrigating the potted seedlings. Water was controlled for five days before salt application to facilitate the diffusion of saline water in dry soil, with 200 mL of salt solution applied per pot during each irrigation. Excess runoff was collected using trays and returned to the pots. Irrigation occurred every two days, with treatments reaching the predetermined soil salinity on the fifth day; the order of irrigation concentrations is provided in **Table 1**. On the day following the attainment of the target salt concentrations, all treatments, except for the control group, were subjected to a gradual drought stress regimen. The watering was controlled to achieve the predetermined moisture gradients using the weighing method daily to replenish the distilled water lost through evaporation. The drought stress duration was calculated from the day after treatment, with samples collected for various parameters on the tenth day post-drought stress (June 25).

**Table 1:** Treatment of soil salinity.

Day/treatment concentration	0.2%NaCl	0.4%NaCl	0.6%NaCl
Day 1	200ml H_2_O	200ml 0.2%NaCl	200ml 0.2%NaCl
Day 3	200ml 0.2%NaCl	200ml 0.2%NaCl	200ml 0.4%NaCl
Day 5	200ml 0.2%NaCl	200ml 0.4%NaCl	200ml 0.6%NaCl
Cumulative salt amount/g	0.8	1.6	2.4
soil salt content (NaCl/Dry soil weight)/%	0.2%	0.4%	0.6%

### 2.2 Measurement indices and methods

#### 2.2.1 Plant growth attributes

After 10-day culture, plant height (for the aboveground part) was measured. The harvested plants were separated into roots and leaves, and the roots were thoroughly washed with tap water to remove any soil. Subsequently, all plant organs were immediately oven-dried at 105°C for 30 min to eliminate any biological activity, followed by further drying at 60°C for 48 h until a constant weight was achieved. The root-to-shoot ratio (R/S) was determined by dividing root biomass by shoot biomass (leaf and stem biomass). The leaf mass ratio (LMR), stem mass ratio (SMR), and root mass ratio (RMR) were calculated as the ratio of leaf biomass to total biomass, stem biomass to total biomass, and root biomass to total biomass, respectively.

#### 2.2.2 Relative water content

According to Turk and Erdal (2015), the leaf relative water content (RWC) was calculated. Immediately following sample collection, the seventh leaf’s fresh weight (FW) was determined. After that, leaf segments were submerged in distilled water for a whole night to calculate their turgid weight (TW). After that, the leaf samples were dried in an oven at 75°C to get their dry weight (DW). The formula RWC = [(FW-DW)/(TW-DW)] × 100 was used to get the RWC.

#### 2.2.3 Lipid peroxidation

Total soluble sugar was estimated in samples using the anthrone sulfuric acid method. Briefly, 0.1 g fresh weight of sample was mixed thoroughly with 10 mL water. Afterwards, the mixture was boiled for 30 min at 100 °C followed by centrifugation. The supernatant was collected and mixed in sulfuric acid-anthrone reagent, then boiled for 10 min at 95-100 °C in a water bath and cooling. The absorption value was read on a spectrophotometer at 620 nm following the method of Zhang et al. (2006), Soluble protein was determined by Coomassie brilliant blue method (Bradford, 1976). 0.5 g samples were ground into a homogenate using 5 mL of 50 mM phosphate buffer (pH 7.8). The homogenate was centrifuged at 12,000 rpm for 20 min. 50 µL of the supernatant was transferred to a test tube and 4 mL Coomassie brilliant blue (0.01%) and 1 mL distilled water were added. After standing for 3 min, absorbance was read at 595 nm in a UV-spectrophotometer, distilled water with Coomassie brilliantblue was used as the blank control. Protein content was determined using the BSA standard curve.

Malondialdehyde (MDA) was determined with thiobarbituric acid (TBA) to analyze the lipid peroxidation (Hodges et al., 1999). The enzyme was extracted from 0.5 g leaf sample by grinding with 5mL 10% trichloroacetic acid (TCA). After 10-min centrifugation at 4000 rpm, 2 mL supernatant liquor was mixed with 2 mL 0.5% TBA, bathed in boiling water for 20 min, cooled down rapidly, and centrifuged at 3000 rpm for 10 min. Spectrophotometry was performed to determine MDA at 450 nm, 532 nm, and 600 nm.

Proline was estimated according to the method of Bates et al. (1973). Fresh leaf samples (0.5 g) were homogenized in 10 mL of sulphosalicylic acid (3% w/v) and the extract was filtered through Whatman’s No. 2 filter paper. In a 2 mL aliquot, 2 mL of acid ninhydrin and 2 mL of glacial acetic acid were added and the contents were boiled for 1 h at 100°C in a water bath. The reaction mixture was further extracted with 2 mL of toluene by mixing thoroughly with vigorous stirring for 15 to 20 sec. Chromophore containing toluene was separated from the aqueous phase. Later absorbance was recordedat 520 nm against toluene blank.

#### 2.2.4 Photosynthetic pigments

Fresh leaf samples (0.1 g) were used for the extraction of pigments in 10 mL of 95% ethanol, which was then centrifuged at 4000 rpm for 10 min. Total chlorophyll, chlorophyll a and chlorophyll b contents were determined according to Lichtenthaler (1987). The absorbance was read at 666, 653 and 470 nm using a spectrophotometer. The amounts of these pigments were calculated and expressed in mg·g^-1^ fresh weight (FW) (Lichtenthaler and Wellburn, 1985).

#### 2.2.5 Analyzing antioxidant enzyme activity

In order to homogenize the leaves (0.2 g), glass powder and a prechilled mortar and pestle were used together with 50 mM K-phosphate buffer (pH 7.0), which contained 1 mM ethylene diamine tetraacetic acid (EDTA) and 1% (w/v) insoluble polyvinyl polypyrrolidone (PVP). A 20 min centrifugation at 4 °C at 15,000 rpm was performed after homogenization. To measure enzyme activity, supernatants were stored at -20 °C (Madamanchi et al., 1994).

As a measure of Catalase (CAT) activity, H_2_O_2_ was degraded over two minutes at 240 nm in the absence of any supernatant. Each enzyme’s specific activity was expressed by the amount of H_2_O_2_ oxidized per mg of protein per minute.

Superoxide dismutase (SOD, U·g^−1^) was assayed using nitroblue tetrazolium (Kamran et al., 2021). The enzyme extract (50 µL) was mixed with 1.5 mL of PBS (0.05 mol·L−1, pH 7.8), 0.3 mL of L-Met (130 mmol·L^−1^), 0.3 mL of NBT (750 µmol·L^−1^), 0.3 mL of EDTA-Na2 (100 µmol·L^−1^) and 0.3 mL of riboflavin (20 µmol·L^−1^). One unit of SOD activity (U·g^−1^) was defined as the amount of enzyme needed to cause 50% inhibition of NBT reduction, as measured at 560 nm.

Peroxidase (POD, U·g^−1^) activity was determined following the guaiacol method (Kamran et al., 2021). The enzyme extract (100 µL) was added to a mixture of 2.9 mL of PBS (50 mmol·L^−1^, pH 7.0), 1 mL of H_2_O_2_ (12%), and 1 mL of guaiacol (50 mmol·L^−1^). Trichloroacetic acid was added after 15 min in a 37 °C water bath to stop the reaction. The absorbance value was recorded at 470 nm.

#### 2.2.6 Abscisic acid

The double antibody sandwich method is used to determine the level of abscisic acid (ABA) in samples. Purified abscisic acid (ABA) antibody is used to coat microplates, creating solid-phase antibodies. Abscisic acid (ABA) is then added to the microplates coated with the primary antibody, followed by the addition of HRP-labeled abscisic acid (ABA) antibody, forming an antibody-antigen-enzyme-labeled antibody complex. After thorough washing, the substrate TMB is added for color development. TMB is converted to blue under the catalysis of HRP and then to the final yellow color under the action of acid. The intensity of the color is positively correlated with the amount of abscisic acid (ABA) in the sample. The absorbance (OD) is measured at a wavelength of 450 nm using an enzyme-linked immunosorbent assay instrument, and the concentration of abscisic acid (ABA) in the sample is calculated using a standard curve.

### 2.3 Data processing

Data organization was conducted using Microsoft Excel, and data analysis was carried out using SPSS version 25.0. Graphs were created using Origin 2024 software from three independent experiments. The significance of differences between data sets was assessed using one-way analysis of variance (ANOVA).

## 3 Results and analysis

### 3.1 Effects of salt and drought stress on the growth of *Viola tricolor* seedlings

Under single salt and drought stress conditions (**Table 2**), with the increase in soil salt concentration and the decrease in water content, plant height, aboveground dry weight, underground dry weight, and root-to-shoot ratio all exhibited a trend of initially increasing and then decreasing. Compared to the control group (CK), the plant height, aboveground dry weight, underground dry weight, and root-to-shoot ratio under the S1D0 treatment increased by 20.92% (*P* < 0.05), 28.42%, 39.81% (*P* < 0.05), and 8.44%, respectively. Under the S0D1 treatment, the increases in plant height, aboveground dry weight, underground dry weight, and root-to-shoot ratio were 4.85%, 17.37%, 33.01% (*P* < 0.05), and 12.84%, respectively. During salt-drought combined stress, when D1 was crossed with different salt concentrations compared to CK, plant height showed a decreasing trend with increasing soil salt concentration. In contrast, the aboveground dry weight, underground dry weight, and root-to-shoot ratio initially increased and then decreased. Under the S1D1 and S2D1 treatments, both aboveground dry weight, underground dry weight, and root-to-shoot ratio were higher compared to CK. Under the D2 treatment crossed with different salt concentrations, plant height, underground dry weight, and root-to-shoot ratio decreased with increasing salt concentration, whereas the aboveground dry weight initially increased and then decreased. In the case of D3 crossed with different salt concentrations compared to CK, plant height, aboveground dry weight, underground dry weight, and root-to-shoot ratio all displayed a decreasing trend with increasing soil salt concentration.

**Table 2:** Changes of plant height,aboveground biomass,underground biomass,root-shoot ratio content in *Viola tricolo*r seedings under salt and drought stress.

Treatment	Plant height/mm	Aboveground biomass/g	Underground biomass/g	Root-shoot ratio
CK	63.42 ± 1.13bc	0.190 ± 0.019abcd	0.103 ± 0.005cd	0.545 ± 0.028abcde
S1D0	77.34 ± 3.31a	0.244 ± 0.013a	0.144 ± 0.013a	0.591 ± 0.042ab
S2D0	59.25 ± 1.71cd	0.177 ± 0.025bcde	0.097 ± 0.011cd	0.557 ± 0.023abcd
S3D0	57.89 ± 0.55d	0.171 ± 0.017bcde	0.091 ± 0.014cdef	0.528 ± 0.028bcdef
S0D1	67.06 ± 0.24b	0.223 ± 0.012ab	0.137 ± 0.006ab	0.615 ± 0.006a
S1D1	63.96 ± 2.08b	0.191 ± 0.016abcd	0.110 ± 0.006bc	0.574 ± 0.020abc
S2D1	64.72 ± 1.96b	0.199 ± 0.019abc	0.113 ± 0.006bc	0.566 ± 0.015abc
S3D1	59.06 ± 0.54cd	0.183 ± 0.011bcd	0.097 ± 0.007cd	0.529 ± 0.013bcdef
S0D2	58.92 ± 1.72cd	0.179 ± 0.021bcde	0.098 ± 0.028cd	0.543 ± 0.025abcde
S1D2	55.84 ± 0.52de	0.193 ± 0.002abcd	0.094 ± 0.007cde	0.487 ± 0.026defg
S2D2	46.16 ± 1.16f	0.126 ± 0.005ef	0.059 ± 0.011gh	0.466 ± 0.033fg
S3D2	40.00 ± 1.16g	0.106 ± 0.030f	0.051 ± 0.027gh	0.476 ± 0.009efg
S0D3	52.77 ± 0.49e	0.124 ± 0.023ef	0.063 ± 0.009fgh	0.512 ± 0.024cdef
S1D3	43.84 ± 2.01fg	0.156 ± 0.023cdef	0.076 ± 0.011defg	0.488 ± 0.016defg
S2D3	42.13 ± 0.56fg	0.140 ± 0.013def	0.064 ± 0.008efgh	0.458 ± 0.019fg
S3D3	40.18 ± 2.98g	0.102 ± 0.004f	0.043 ± 0.000h	0.425 ± 0.009g

(Different lowercase letters indicate significant differences among treatments at the 0.05 level. This applies to the following figures as well).

### 3.2 Effects of salt and drought stress on the relative water content of *Viola tricolor* seedling leaves

**Figure 1:**
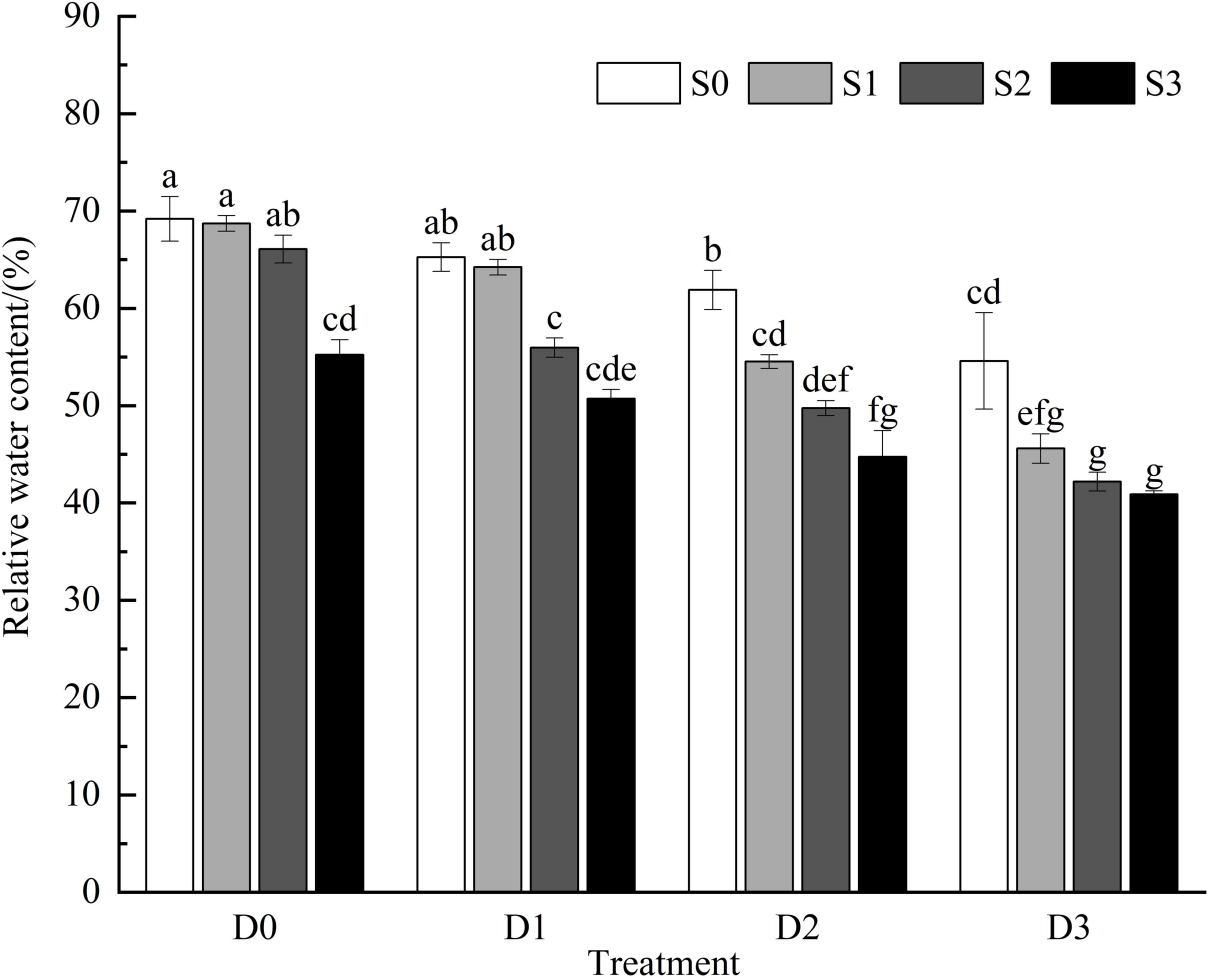
Changes of relative water content in *Viola tricolor* seedings under salt and drought stress. (Different lowercase letters indicate significant differences among treatments at the 0.05 level. This applies to the following figures as well).

Research indicates that under single salt stress and single drought stress conditions (**Figure 1**), the relative water content of *Viola tricolor* leaves tends to decrease as the stress intensity increases. There were no significant differences in relative water content among the S1D0, S2D0, and S0D1 treatments compared to the control group (CK) (*P* > 0.05). However, the S3D0, S1D2, and S0D3 treatments significantly reduced the relative water content of the Viola tricolor seedlings (*P* < 0.05). During salt-drought combined stress, when D1 was crossed with different salt concentrations, the relative water content showed a decreasing trend with increasing soil salt concentration compared to CK, and the decrease under the S1D1 treatment was insignificant (*P* > 0.05). In contrast, under the S2D1 and S3D1 treatments, the relative water content decreased significantly by 19.12% and 26.69%, respectively (*P*< 0.05). Under the D2 and D3 treatments crossed with different salt concentrations, the relative water content of Viola tricolor seedling leaves also exhibited a decreasing trend with increasing soil salt concentration, showing significant differences compared to CK (*P* < 0.05).

### 3.3 Effects of salt and drought stress on MDA content and osmotic adjustment substances in *Viola tricolor* seedlings

**Figure 2:**
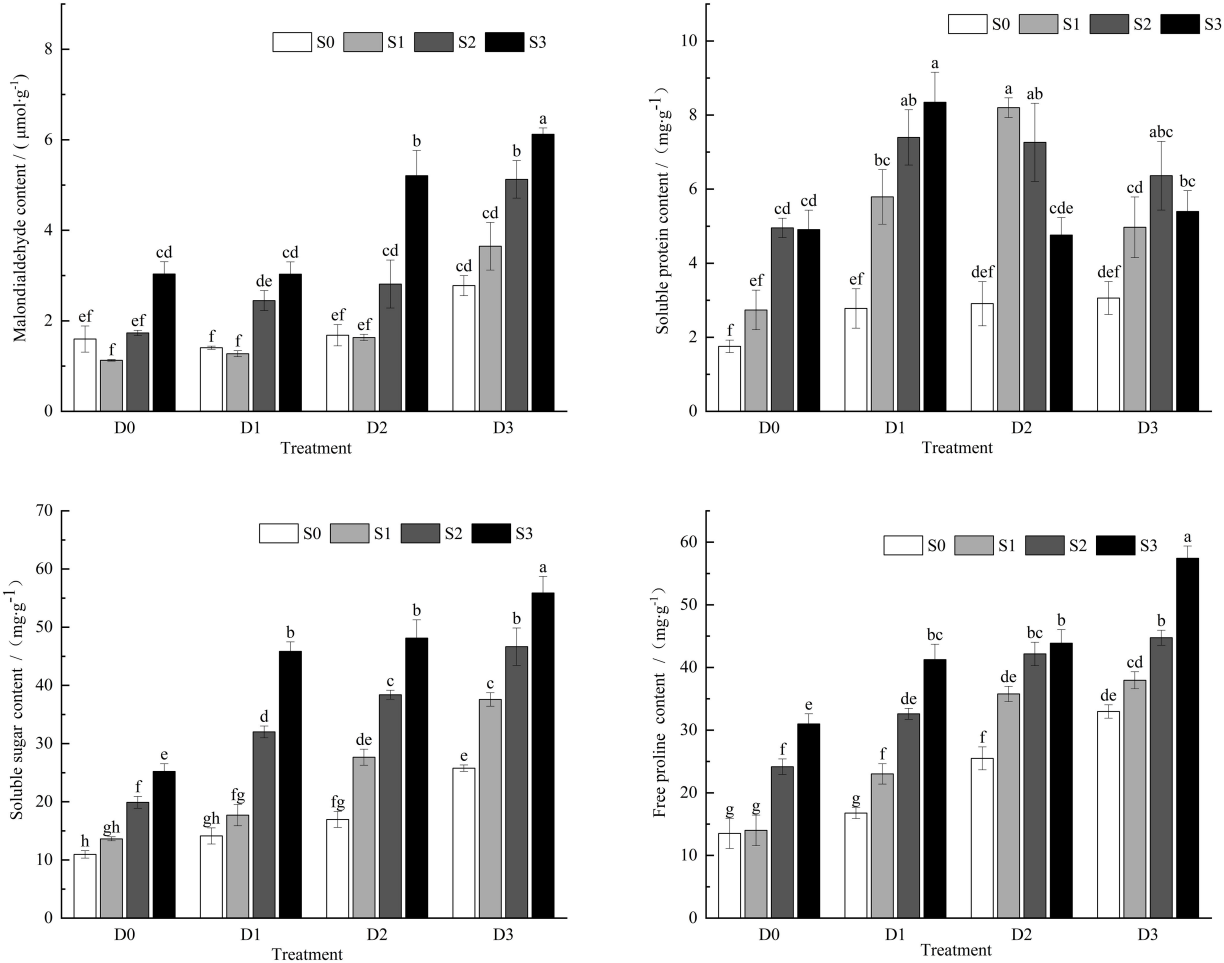
Changes of MDA,SP,SS,Pro content in *Viola tricolor* seedings under salt and drought stress.

Under single salt and drought stress conditions (**Figure 2**), the MDA content in the leaves of *Viola tricolor* seedlings exhibited a trend of initially decreasing and then increasing with the rise in soil salt concentration and the decline in water content. In contrast, the contents of soluble proteins, sugars, and proline showed an increasing trend. During salt-drought combined stress, under the D1 treatment crossed with different salt concentrations, the MDA content demonstrated a pattern of initially decreasing and then increasing, with a decrease of 20.30% compared to CK under the S1D1 treatment. The S2D1 and S3D1 treatments significantly increased MDA content (*P* < 0.05), with increases of 53.27% and 89.90%, respectively. The soluble protein, sugar, and proline levels continued to increase significantly (*P* < 0.05). Under the D2 treatment crossed with different salt concentrations, MDA content initially decreased and then increased with the rising soil salt concentration. At the same time, soluble protein content first increased and then decreased, and soluble sugars and proline contents exhibited an increasing trend. In the D3 treatment with different salt concentrations, both MDA content and the soluble sugars and proline levels increased with the rising soil salt concentration. In contrast, the soluble protein content showed an initial increase followed by a decrease.

### 3.4 Effects of salt and drought stress on the activity of antioxidant enzymes in *Viola tricolor* seedlings

**Figure 3:**
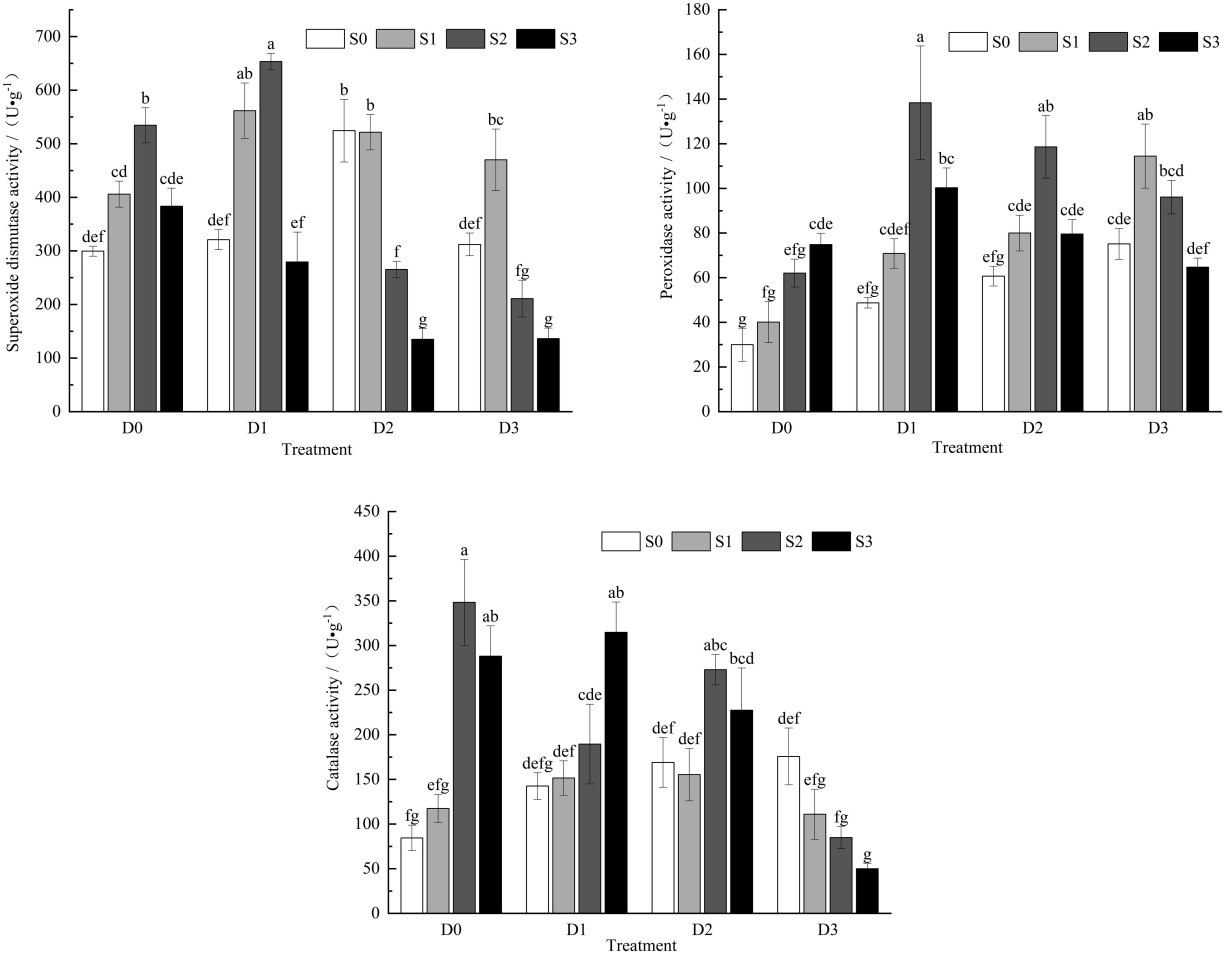
Changes of CAT, POD, SOD activity in *Viola tricolor* seedlings under salt and drought stress.

Under single salt stress conditions (**Figure 3**), the activities of SOD and CAT in the leaves of the control group (CK) increases were 35.51%, 78.42%, and 28.02% for SOD activity and 7.17%, 75.12%, and 4.19% for CAT activity, respectively. The activity of POD showed an increasing trend, with enhancements of 33.79%, 106.9%, and 149.5% compared to CK. Under single drought stress, the activities of POD and CAT increased with the intensification of soil drought, showing 62.5%, 102.3%, and 150.4% for POD and 68.6%, 99.96%, and 107.9% for CAT, respectively. SOD activity exhibited a trend of initially increasing and then decreasing, with the S0D2 treatment significantly increasing SOD activity in *Viola tricolor* seedlings (*P* < 0.05). During salt-drought combined stress, under the D1 treatment crossed with different salt concentrations, SOD and POD activities initially increased and then decreased with the increase in soil salt concentration. Both activities reached their maximum under the S2D1 treatment, showing significant differences compared to CK (*P*< 0.05), while CAT activity exhibited an increasing trend, with significant differences compared to CK (*P* < 0.05). Under the D2 treatment crossed with different salt concentrations, SOD, POD, and CAT activities showed a trend of initially increasing and then decreasing with increasing soil salt concentration compared to CK. In the D3 treatment crossed with different salt concentrations, SOD and POD activities increased initially and then decreased with rising soil salt concentration. Under the S2D3 and S3D3 treatments, SOD activity decreased by 29.55% and 59.51% compared to CK, respectively, while POD activity increased by 281.4%, 220.3%, and 115.7%. In contrast, CAT activity decreased, reaching its lowest point under the S3D3 treatment, with a reduction of 40.79% compared to CK.

### 3.5 Effects of salt and drought stress on chlorophyll content in *Viola tricolor* seedlings

**Figure 4:**
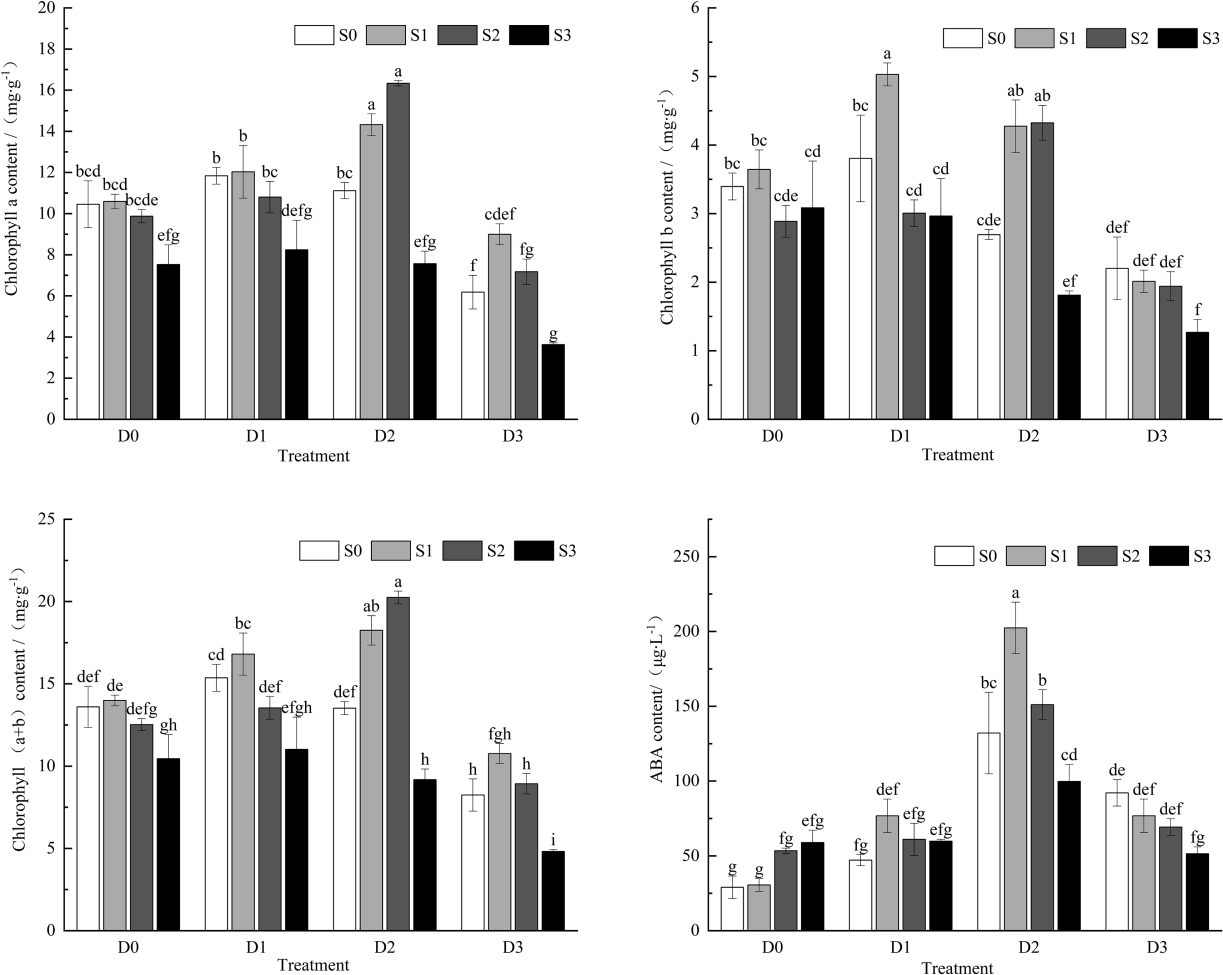
Changes of chla, chlb, chl (a+b), ABA content in *Viola tricolor* seedings under salt and drought stress.

Under single salt and drought stress conditions (**Figure 4**), as the stress intensity increased, the contents of chlorophyll a, chlorophyll b, and total chlorophyll exhibited a trend of initially increasing and then decreasing. During salt-drought combined stress, under the D1 treatment crossed with different salt concentrations, the contents of chlorophyll a, chlorophyll b, and total chlorophyll initially increased and then decreased with the increase in soil salt concentration. The maximum chlorophyll b content was observed under the S1D1 treatment, which showed a significant difference compared to the control group (CK) (*P* < 0.05). Under the D2 treatment crossed with different salt concentrations, chlorophyll a, chlorophyll b, and total chlorophyll contents also exhibited a trend of initially increasing and then decreasing as soil salt concentration increased, with chlorophyll a and total chlorophyll reaching their highest values under the S2D2 treatment, significantly different from CK. In the S3D2 treatment, chlorophyll a, b, and total chlorophyll contents were significantly lower than those in CK (*P* < 0.05). Under the D3 treatment crossed with different salt concentrations, the contents of chlorophyll a, chlorophyll b, and total chlorophyll decreased with increasing soil salt concentration compared to CK. Compared to the single drought treatment (S0D3), chlorophyll a and total chlorophyll contents exhibited a trend of initially increasing and then decreasing. All chlorophyll contents (chlorophyll a, chlorophyll b, and total chlorophyll) reached their lowest values under the S3D3 treatment, showing significant reductions of 65.26%, 62.83%, and 64.56%, respectively, compared to CK (*P* < 0.05).

### 3.6 Effects of salt and drought stress on abscisic acid content in *Viola tricolor* seedlings

Under single salt stress conditions (**Figure 4**), Viola tricolor seedlings’ abscisic acid (ABA) content exhibited an increasing trend with an increase in soil salt concentration. Under single drought stress conditions, the ABA content initially increased and then decreased as soil salt concentration increased. During salt-drought combined stress, under the D1 and D2 treatments crossed with different salt concentrations, the ABA content in Viola tricolor seedlings initially increased and then decreased as the stress intensity increased. The ABA content was higher under the moderate drought condition of D2 than other drought treatments, reaching its maximum value under the S1D2 treatment. It was significantly greater than CK and the treatments under single drought and salt stress. The ABA content decreased with increased soil salinity under the D3 treatment crossed with different salt concentrations. Compared to the S0D3 treatment, the ABA contents decreased by 16.66%, 24.81%, and 44.12% under the S1D3, S2D3, and S3D3 treatments, respectively, but were still higher than that of CK.

### 3.7 Correlation analysis of Growth and Physiological parameters in *Viola tricolor* seedlings under salt and drought stress

**Figure 5:**
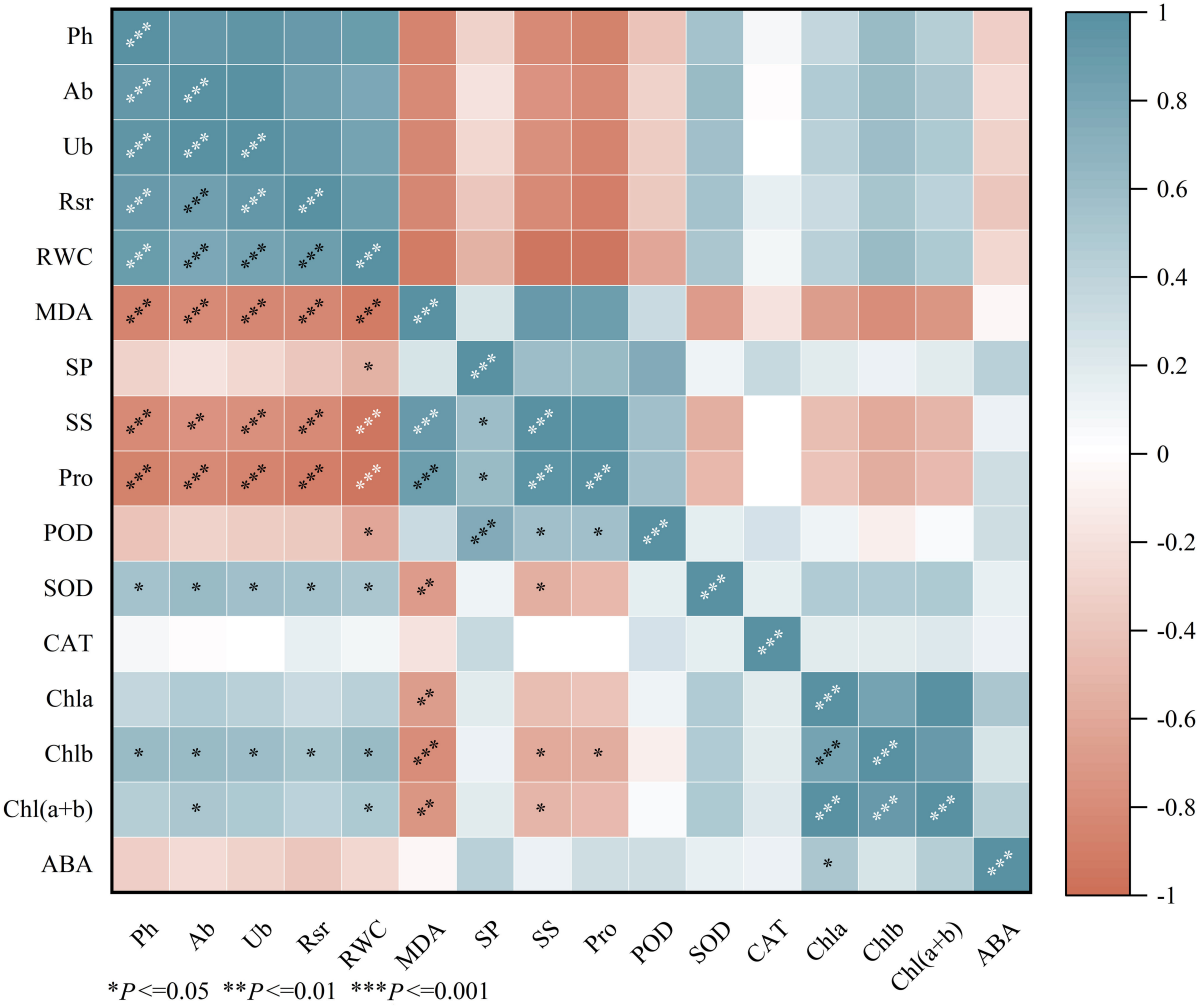
The correlation heat map. (Ph, Plant height; Ab, Aboveground biomass; Ub, Underground biomass; Rsr, Rootshoot ratio; RWC, Relative water content; MDA, Malondialdehyde; SP, Soluble Protein; SS, Soluble Suger; Pro, proline; POD, Peroxidase; SOD, Superoxide dismutase; CAT, Catalase; Chla, Chlorophyll a; Chlb, Chlorophyll b; Chl(a+b), Chlorophyll (a+b); ABA, Abscisic acid).

The results of the correlation analysis (**Figure 5**) indicate that the plant height, aboveground biomass, underground biomass, root-to-shoot ratio, relative water content, SOD activity, and chlorophyll b (Chlb) content of *Viola tricolor* seedlings showed significant positive correlations (*P*<0.05). In contrast, they exhibited significant negative correlations with malondialdehyde (MDA), soluble sugars (SS), and proline (Pro) contents (*P* < 0.05). MDA was found to have a highly significant negative correlation with SOD, chlorophyll a (Chla), chlorophyll b (Chlb), and total chlorophyll (Chl (a+b)) (*P* < 0.01). Peroxidase (POD) showed a significant negative correlation with relative water content (RWC) (P < 0.05) and significant positive correlations with soluble proteins (SP), SS, and Pro (*P* < 0.05). Additionally, ABA exhibited a significant positive correlation with Chla (*P*<0.05).

### 3.8 PCA of growth and physiological indicators in *Viola tricolor* seedlings under salt and drought stress

**Figure 6:**
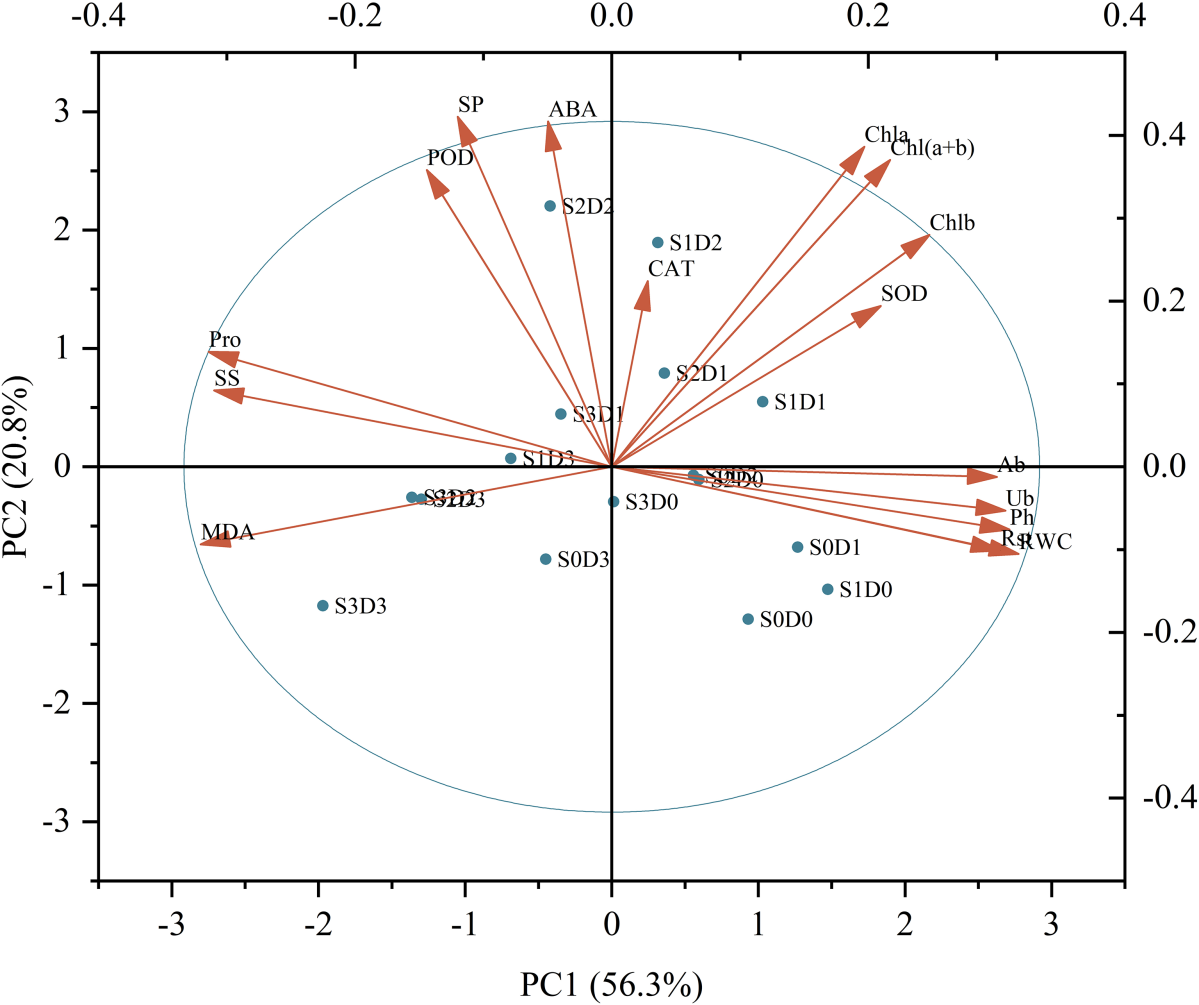
Principal component analysis among the indicators measured of Viola tricolour seedlings. (Ph, Plant height; Ab, Aboveground biomass; Ub, Underground biomass; Rsr, Rootshoot ratio; RWC, Relative water content; MDA, Malondialdehyde; SP, Soluble Protein; SS, Soluble Suger; Pro, proline; POD, Peroxidase; SOD, Superoxide dismutase; CAT, Catalase; Chla, Chlorophyll a; Chlb, Chlorophyll b; Chl(a+b), Chlorophyll (a+b); ABA, Abscisic acid).

Principal component analysis (PCA) was conducted to assess *Viola tricolor* seedlings’ growth and physiological indicators subjected to salt and drought stress treatments (**Figure 6**). The results indicate that the first axis accounted for 56.3% of the total variance, primarily determined by parameters such as pH (Ph), abscisic acid (Ab), urea (Ub), root strength (Rst), relative water content (RWC), malondialdehyde (MDA), soluble sugars (SS), and proline (Pro). The second axis explained 20.8% of the total variance, mainly influenced by soluble proteins (SP), chlorophyll a (Chla), chlorophyll b (Chlb), total chlorophyll (Chl (a+b)), and abscisic acid (ABA). The third axis accounted for 8.3% of the variance, primarily determined by peroxidase (POD), superoxide dismutase (SOD), and catalase (CAT). Collectively, these three dimensions explained 85.3% of the total variance of all sixteen traits. This study employed a membership function method to qualitatively evaluate the damage caused by single salt, drought, and salt-drought combined stresses on Viola tricolour seedlings. From **Table 3**, it is evident that the comprehensive scores for the membership function under salt-drought treatment ranked as follows: S1D1 > S1D0 > S0D1 > S2D1 > S1D2 > S2D0 > S0D2 > S0D0 (CK) > S2D2 > S3D1 > S3D0 > S1D3 > S0D3 > S2D3 > S3D2 > S3D3. The ranking indicates that Viola tricolor seedlings exhibit a specific salt and drought tolerance level under mild and moderate single salt and drought stress. Under combined salt-drought stress, seedlings treated with S1D1, S2D1, and S1D2 also demonstrated a degree of tolerance, with the highest membership function value observed for the S1D1 treatment, indicating the most robust salt and drought resistance.

**Table 3:** Comprehensive evaluation of the tolerance of *Viola tricolor* leaves under cross stress of salt and drought.

Treatment	PC1	PC2	PC3	Score	Ranking
S0D0	0.842	0.000	0.000	0.556	8
S0D1	0.941	0.174	0.290	0.691	3
S0D2	0.734	0.348	0.135	0.582	7
S0D3	0.441	0.145	0.254	0.351	13
S1D0	1.000	0.072	0.402	0.717	2
S1D1	0.871	0.526	0.337	0.736	1
S1D2	0.663	0.911	0.040	0.663	5
S1D3	0.372	0.389	0.473	0.386	12
S2D0	0.745	0.338	0.648	0.636	6
S2D1	0.676	0.596	1.000	0.688	4
S2D2	0.450	1.000	0.030	0.543	9
S2D3	0.195	0.290	0.376	0.236	14
S3D0	0.576	0.284	0.612	0.509	11
S3D1	0.471	0.496	0.922	0.521	10
S3D2	0.176	0.295	0.246	0.212	15
S3D3	0.000	0.032	0.279	0.035	16

## 4 Discussion

Growth parameters of plants are the most direct indicators reflecting the extent of damage sustained by the plants. Biomass represents a comprehensive reflection of plant growth under stress conditions. It is also an important indicator of plant viability (Tang et al., 2015). In another studies, Yu et al. (2023) on *Iris japonica* and Guo et al. (2022) on *Asparagus* found that drought stress decreased the growth of plants, and more growth reduction was found in leaves than that in roots under salt stress. However, plants respond differently to varying intensities of stress. This study indicates that under mild salt stress, drought stress, and mild to moderate salt-drought combined stress, the plant height, aboveground dry weight, underground dry weight, and root-to-shoot ratio of Viola tricolor seedlings were all higher than those under control conditions. The seedlings exhibited a degree of cross-adaptation under mild to moderate salt stress. As the concentration increased, however, the plant height, aboveground dry weight, underground dry weight, and root-to-shoot ratio of the seedlings were continually suppressed under moderate to severe salt-drought stress and their combined effects. The result is in line with other studies (Shamuyarira et al., 2022; Liu et al., 2022), indicating that biomass accumulation and allocation patterns can be altered to acclimate to salt and drought, under low-concentration stress conditions, Viola tricolor seedlings preferentially allocate more resources to their root systems, allowing them to absorb more water and nutrients, thereby reducing the detrimental effects of water deficiency on both roots and leaves through an increased root-to-leaves ratio.

The relative water content of leaves is the basic material used to maintain plants’ normal growth and physiological and metabolic activities in an orderly manner, and its content can reflect the degree of damage caused by salt and drought stress on plants (Dai et al., 2021). It has been shown that salt and drought stress can significantly reduce tissue water content in plant seedlings (Weng et al., 2017). In this study, we found that salt stress, drought stress and cross-stress of the two can reduce the leaf water content of pansy seedlings; under mild salt, drought and cross-stress of the two, the difference between the leaf water content of pansy seedlings and the control treatment is not significant. The change is small, which indicates that the leaf tissue of pansy seedlings has a certain degree of anti-dehydration ability. It can maintain the normal operation of physiological and biochemical activities by reducing the loss of water and increasing the water utilization efficiency, while with the increase of stress concentration, the soil salt content and soil water content have exceeded the tolerance range of the plant. Normal operation of physiological and biochemical activities to make it grow normally in adversity, and with the increase of stress concentration, soil salinity and soil water content has exceeded the tolerance range of pansy seedlings, the plant must maintain the cell expansion pressure by reducing the water potential of the leaves to reduce its injury, which is in line with the results of the studies by Zheng et al. (2020), It also suggests that reducing leaf water content is an effective osmoregulatory mechanism carried out by plants to resist stress.

MDA is a physiological indicator of the degree of peroxidation of membrane lipids under adverse conditions in plants, and its level reflects the degree of peroxidative damage of the plasma membrane (Jafarnia et al., 2018). The content increases, manifested by increased cell membrane permeability, which hinders the normal metabolic activities of cells (Didi et al., 2022). Studies (Lan et al., 2022) have shown that salt and drought stress disrupts the cell membrane integrity of overlord seedlings, increasing MDA content. This study found that under mild salt, drought, and combined stress conditions, the malondialdehyde (MDA) content was lower than in the control treatment. It is hypothesized that mild salt-drought stress may weaken lipid peroxidation, resulting in a correspondingly lower MDA content and less damage to the membrane system. However, as the intensity of salt and drought stress increased, MDA levels continuously rose, indicating that the accumulation of free radicals within Viola tricolor led to lipid peroxidation, causing severe damage to the cell membranes. This finding is similar to the results reported by Yu et al. (2021) on the seedlings of sand reed grass. Soluble sugars, proteins, and proline act as osmotic regulators, mitigating the damage caused by stress in plants under adverse conditions (Yang et al., 2024a). This study indicates that with the increasing severity of single salt stress, drought stress, and their combined salt-drought stress, the contents of soluble sugars, soluble proteins, and proline all exhibited an upward trend, with a more pronounced increase under the salt-drought combined treatment. This suggests that Viola tricolor seedlings can effectively enhance their cellular osmotic pressure and lower cellular water potential by increasing the levels of osmotic regulators under salt and drought stress, thereby alleviating cellular damage and promoting plant growth. These findings are consistent with those reported by Li et al. (2022) and Öztürk et al. (2020).

SOD, POD, and CAT are critical enzymes in the plant’s antioxidant scavenging system (Foyer and Noctor, 2005). SOD serves as an important free radical scavenging enzyme, catalyzing the dismutation of superoxide radicals (·O_2_) into hydrogen peroxide (H_2_O_2_) and oxygen (O_2_), thus alleviating the toxic effects of superoxide anions on plants (Huo et al., 2022). The resulting H_2_O_2_ can lead to lipid peroxidation as a reactive oxygen species (ROS). CAT and POD primarily scavenge H_2_O_2_ from various cellular locations, which is the product of SOD-catalyzed reactions involving ·O_2_ (Asada, 1999). Thus, the synergistic action of SOD, CAT, and POD is necessary to defend against the oxidative stress caused by the excessive accumulation of reactive oxygen species. Different plants utilize various enzymes to protect themselves from environmental stresses. The results of this study indicate that under single salt and drought stress conditions, the activities of POD, CAT, and SOD in Viola tricolor seedlings overall exhibited an increasing trend, although, under severe salt and drought stress, SOD activity decreased yet remained higher than that of the control treatment. This finding is consistent with Kong et al. (2017). Zhuang et al. (2010) found that under low to moderate salt-drought combined stress, the antioxidant enzyme activities in *Ammodendron argenteum* seedlings increased, while under severe stress, the activities of these enzymes were significantly lower than in control. This study has shown that as the severity of salt-drought combined stress increases, the activities of SOD, POD, and CAT in Viola tricolor seedlings displayed a trend of first increasing and then decreasing. This indicates that under light to moderate salt and drought combined treatments, the enzymes responsible for scavenging free radicals in Viola tricolor seedlings are SOD, POD, and CAT. However, under severe salt (0.6% NaCl) and drought (35% field capacity) treatment, POD activity remained significantly higher than that of the control (*P* < 0.05), suggesting that POD plays a predominant role in the reactive oxygen species scavenging system under high salt and severe drought conditions. This also implies that protective enzyme activities can eliminate free radicals produced within plants up to a particular stress threshold, reducing damage to cell membranes. However, when the stress exceeds its maximum capacity, the dynamic balance between generating and eliminating ROS is disrupted, leading to decreased enzyme activity. This finding is similar to the results obtained by Wang et al. (2018).

Chlorophyll plays a critical role in plants’ primary processes of energy absorption, conversion, and transfer. It is positively correlated with the rate of photosynthesis within a specific range (Agastian et al., 2000). This study indicates that the contents of chlorophyll a, chlorophyll b, and total chlorophyll exhibit an increasing trend under mild salt and drought stress However, under moderate and severe salt and drought stress conditions, the levels of chlorophyll a, b, and total chlorophyll in Viola tricolor seedlings were significantly lower than those in the control treatment. Under salt-drought combined stress, the chlorophyll content generally showed a trend of first increasing and then decreasing. The chlorophyll content was higher than the controls at mild to moderate salt-drought combined stress levels. In contrast, under severe salt stress and various drought stress combinations, as well as under severe drought and varying salt stress combinations, the content of chlorophyll a, chlorophyll b, and total chlorophyll were all suppressed. This may be because Viola tricolor enhances its chlorophyll content to alleviate mild stress; however, as stress intensifies, its capacity for alleviation declines, resulting in reduced chlorophyll levels. This observation aligns with the findings of Hu et al. (2022).

Abscisic acid (ABA) is one of the essential endogenous hormones in plants and serves as the most critical regulatory substance for plant responses to abiotic stress. Under normal conditions, plants can alleviate damage by increasing their internal ABA levels in response to stress (Kuromori et al., 2018; Ullah et al., 2018). Previous studies have shown that salt and drought stress can rapidly increase endogenous ABA levels (Farooq et al., 2011). This study indicates that ABA levels show an overall increasing trend under single salt and drought stress; however, a decline in ABA levels is observed under severe drought stress. Under salt-drought combined stress, ABA content increases and decreases under mild and moderate drought combined with varying salt concentrations. In contrast, ABA content shows a decreasing trend under severe drought combined with different salt concentrations. This decline may be due to the severe damage sustained by the plant in a highly drought-stressed environment, leading to reduced root activity, which weakens the synthesis and transport capacity of ABA, resulting in lower ABA levels in the leaves (Zhao et al., 2024).

## 5 Conclusion

Drought, salt stress, and salt-drought combined stress all significantly affect *Viola tricolor* seedlings’ growth and physiological characteristics to varying degrees. Plant height, biomass, antioxidant enzyme activity, chlorophyll content, and abscisic acid content initially increased and then decreased with the intensification of salt-drought stress. At the same time, the contents of MDA and osmotic adjustment substances exhibited an increasing trend. *Viola tricolor* seedlings enhance their adaptability to saline-drought environments and mitigate damage by regulating these physiological indicators. The seedlings demonstrate a certain tolerance level to moderate and low levels of salt-drought combined stress, showing signs of cross-adaptation. The most robust salt and drought tolerance is observed under the treatment of 0.2% NaCl combined with 65% field capacity of mild drought. POD emerges as the primary antioxidant enzyme in *Viola tricolor* seedlings when faced with high-salt and severe drought stress. These findings hold theoretical and practical significance for evaluating the stress resistance of the *Viola tricolor* and expanding its cultivation.

## Data Availability

The original contributions presented in the study are included in the article/supplementary material. Further inquiries can be directed to the corresponding author.
